# Immunoadsorption treatment for dilated cardiomyopathy: A PRISMA-compliant systematic review and meta-analysis

**DOI:** 10.1097/MD.0000000000026475

**Published:** 2021-07-02

**Authors:** Ru-tao Bian, Zhen-tao Wang, Wei-yu Li

**Affiliations:** a Department of Cardiology, Henan University of Chinese Medicine; b Department of Cardiology, The Second Affiliated Hospital of Henan University of Chinese Medicine; c Department of Nephropathy, Zhengzhou Traditional Chinese Medicine Hospital, Zhengzhou, China.

**Keywords:** dilated cardiomyopathy, immunoadsorption, meta-analysis

## Abstract

**Background:**

As one of the leading causes of heart failure, dilated cardiomyopathy (DCM) is characterized by dysfunctional muscle contraction and enlarged ventricular chamber. Patients with DCM have been shown to respond well to immunoadsorption (IA) therapies. However, the efficacy and safety of IA treatment for DCM patients remained to be evaluated.

**Methods:**

This study was designed in accordance with the Preferred Reporting Items for Systematic Review and Meta-analysis. We searched the databases such as Cochrane library, Cochrane Central Register of Controlled Trials, Embase, OVID, and Web of Science from January 1990 to March 20, 2020, and performed meta-analysis using Stata MP Version 13.0.

**Results:**

We performed meta-analysis on 12 studies that included a total of 395 patients with DCM. Overall, IA treatment significantly improved the left ventricular ejection fraction (6.01, 95% confidence interval [CI] [4.84–7.19]), reduced the left ventricular end diastolic diameter (–3.62, 95% CI [–4.06 to –3.19]), reduced severity of symptoms according to the New York Heart Association (NYHA) functional classification (–1.37, 95% CI [–1.73 to –1.02]) as compared with the controls, but had no effect on values for safety parameters (1.13, 95% CI [0.58–2.19]).

**Conclusions:**

Results of this meta-analysis indicated that the IA treatment can improve the left ventricular ejection fraction, reduce left ventricular end diastolic diameter, and thus improve clinical outcome in DCM patients. However, further evidence are required to validate the relative safety of IA treatment. Multi-center, double blind studies should be conducted to elucidate the precise effect of IA treatment in DCM patients.

## 1 Introduction

Dilated cardiomyopathy (DCM) refers to impaired left ventricles that cause dilatation and systolic dysfunction in the absence of abnormal loading conditions or coronary artery disease.^[1]^ Moreover, DCM can lead to end-stage heart failure which is the most frequent indication for heart transplant worldwide.^[2]^ The pathogenesis of DCM is multifactorial. Inherited DCM is caused by genetic variants, whereas acquired DCM is caused by one or multiple factors including toxic agents, viral infection, immune disorders, hormonal changes, and arrhythmias.^[3]^ DCM is more common in men than in women and has higher mortality in men. The prevalence of DCM is 40 in 100,000 persons with an annual incidence of 7 in 100,000 persons.^[4]^ Great advances has been made in the treatment of DCM through prevention of heart failure or concomitant adverse events. However, rehospitalization and mortality of DCM patients remain high.^[5]^

Recent studies on DCM suggest that anti-myocardial autoantibodies can trigger or aggravate myocardial contractile dysfunction.^[6]^ Programmed cell death protein 1 is a negative immunoregulatory receptor that is expressed on the surface of T lymphocytes. In programmed cell death protein 1-deficient mice, immunoglobulin G (IgG) was found to be deposited on the surface of cardiomyocytes, suggesting that the autoimmune system is involved in the disease progress of DCM.^[7]^ In DCM patients, removal of antibodies by immunoadsorption (IA) can improve hemodynamic. Immunoadsorption and subsequent intravenous immunoglobulin (IVIG) substitution (IA/IgG) can improve left ventricular ejection fraction (LVEF) and endothelial function, relieve DCM symptoms, and increase cardiac index.^[8]^ Accordingly, many studies have evaluated the safety and efficacy of IA in DCM patients.^[9]^ However, these randomized controlled trials have small sample sizes and weak statistical power. Therefore, meta-analysis of available studies is required to assess the efficacy of IA treatment on DCM patients.

## 2 Methods

The meta-analysis was conducted according to Cochrane Handbook guidelines and the Preferred Reporting Items for Systematic Review and Meta-analysis statements. The protocol for this review was registered with PROSPERO (CRD42020182510). No ethical guidelines were required or applied in this study.

### 2.1 Search strategy

We systematically searched Cochrane library, Cochrane Central Register of Controlled Trials, Embase, OVID, and Web of Science from January 1990 to March 20, 2020. To avoid omitting any related publication, we also screened the www.clinicaltrials.gov. Search terms used in this study include “Cardiomyopathy, Dilated [MeSH],” “Plasmapheresis [MeSH],” and “dilated cardiomyopathy,” “Immunoadsorption,” “Immunoglobulin,” “IgA/IgG.” Summary and quotations of studies were reviewed by 2 authors.

### 2.2 Study selection

We included research articles that: investigated DCM patients; was published in English; included 2 comparable groups, of which one received IA or IA/IG and IVIG therapy; measured LVEF, left ventricular end diastolic diameter (LVEDD), adverse events or the NYHA classification; followed up patients for at least 1 month. We excluded research and review studies that was designed in an analytical, observational, open, or retrospective manner; used animal models.

### 2.3 Extraction of data and assessment of quality

R-tB and W-yL, independently extracted first author, publication year, sample size, and other data of interest from individual studies. Disagreement, if any, was resolved after discussion between the 2 authors. If required, a third author was consulted. GetData Graph Digitizer 2.25 (http://getda ta-graph -digit izer.com) was used to generate graphs from the results. In addition, to validate the appraised study, we used risk of bias tool as recommended by the Cochrane Collaboration. Studies were assessed for blinding, selective outcome reporting, description of follow-ups, and other threats to validity.

### 2.4 Statistical analysis

The statistical software Stata MP Version 13.0 (StataCorp, College Station, TX) was used to carry out the meta-analysis. Continuous variable was analyzed using the standard mean difference, or the weighted mean difference, and 95% confidence intervals (CI). For dichotomous data, CI and risks ratio were calculated. Otherwise, standard deviations were calculated according to the Cochrane Handbook for Systematic Reviews of Interventions.^[10]^ Cochrane *Q* test and *I*^2^ were used to evaluate the heterogeneity of each study, and a *P* < .10 was considered as significant heterogeneity. When the value of *I*^2^ exceeds 50%, the corresponding study was considered as significant heterogeneity among the trials and a random-effect model was used. When the value of *I*^2^ was under 50%, results were analyzed using fixed effect model. In addition, a subgroup analysis was conducted in order to find out whether the LVEF efficacies were consistent in IA or IA/IG and IVAG-treated group. In order to objectively evaluate the publication bias, we used the Stata MP version 13.0 software to generate the funnel plot (n ≥ 10 studies) and perform the Egger test. *P* < .05 was considered as the existence of publication bias.

## 3 Results

**Figure 1:**
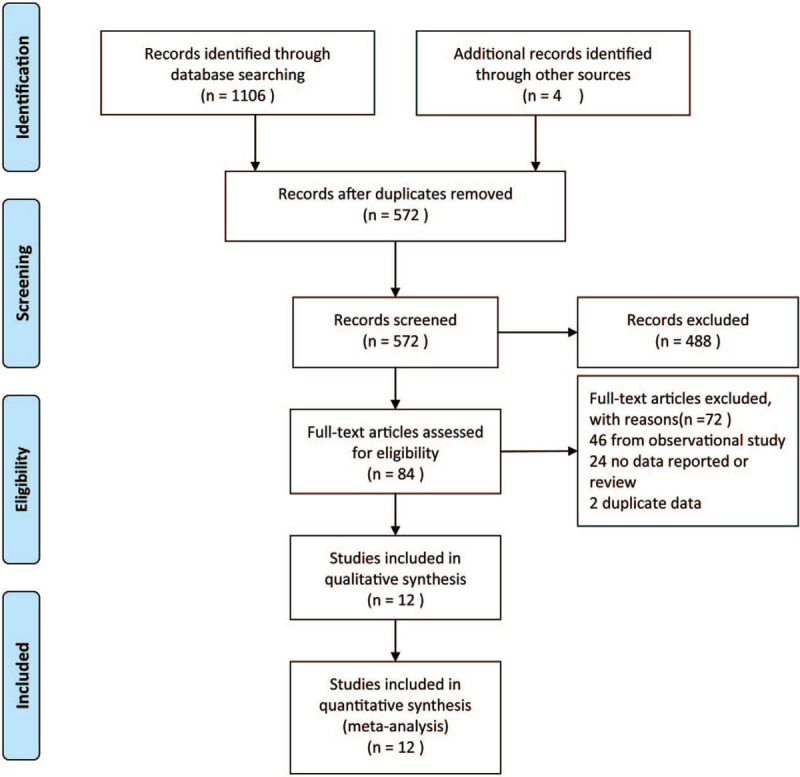
Flow chart of database search and literature identification.

The database search revealed 1110 studies, after removal of 538 duplicated studies, and 488 irrelevant records were excluded based on abstracts and titles. According to the inclusion criteria, a total of 84 publications were retrieved, 70 were excluded as they were reviews, designed inappropriately or did not include related drugs, and 2 articles duplicated data with other studies. Finally, 12 studies were amenable for inclusion in the meta-analysis (Fig. 1).

### 3.1 Study characteristics

This meta-analysis investigated 395 DCM patients including 201 patients that received IA therapy and 194 that received optimal medical treatments other than IA. Key characteristics of all included studies are listed in Table 1. These studies were published between the years of 2000 and 2013, and the age range of patients was from 43 to 60.5 years. Among these studies, 5 studies assessed IA therapy,^[11–15]^ 4 studies assessed IA/IgG polytherapy,^[16–19]^ 3 studies assessed IVIG,^[20–22]^ and 2 studies used placebo treatment in the control group.^[20,22]^

**Table 1:**
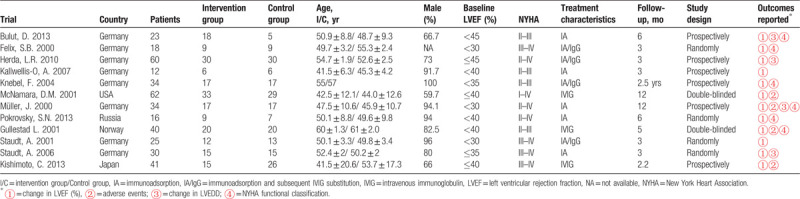
Characteristics of the included clinical trials.

### 3.2 Data quality

**Figure 2:**
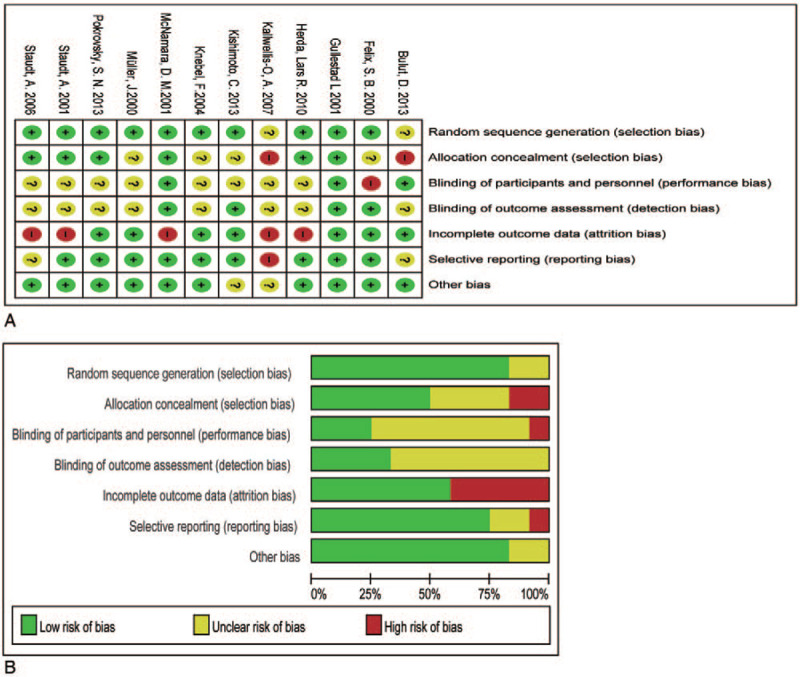
Risk of bias assessment for studies. (A) Details of quality evaluation for each of the included in studies; (B) summary of risk of bias of the included in the meta-analysis.

Key details regarding risks of biases as recommended by the Cochrane Collaboration tool are listed in Fig. 2. Among the 12 included studies, 6 were prospective studies, 2 were double-blinded studies, and 4 were random studies.

### 3.3 Quantitative synthesis

#### 3.3.1 Left ventricular ejection fraction

**Figure 3:**
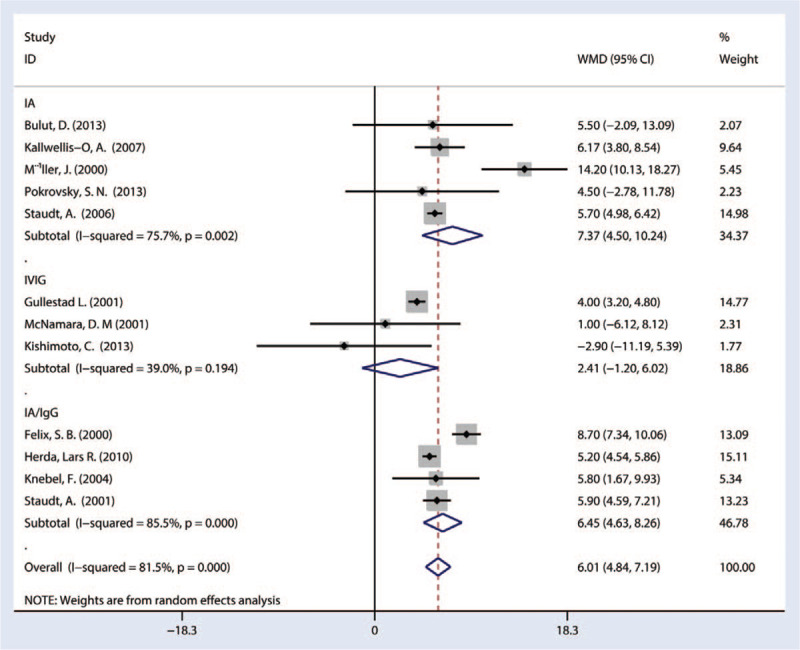
Forest plot showing the LVEF immunoadsorption treatment in patients of DCM. DCM = dilated cardiomyopathy, LVEF = left ventricular ejection fraction.

Combined results from 12 studies showed that implementation of IA therapy significantly improved LVEF as compared with that of control (6.01, 95% CI [4.84–7.19]) with significant heterogeneity (*P* = 0.001, *I*^*2*^ = 81.5%). Subgroup stratified by the test showed that only IA therapy significantly improved the LVEF (7.37, 95% CI [4.50–10.24]), and that the IVIG only therapy had no significant effect on the LVEF (2.41, 95% CI [–1.20–6.02]), and IA/IgG treatment significantly improved the LVEF (6.45, 95% CI [4.63–8.26]) (Fig. 3).

#### 3.3.2 Left ventricular end diastolic diameter

**Figure 4:**
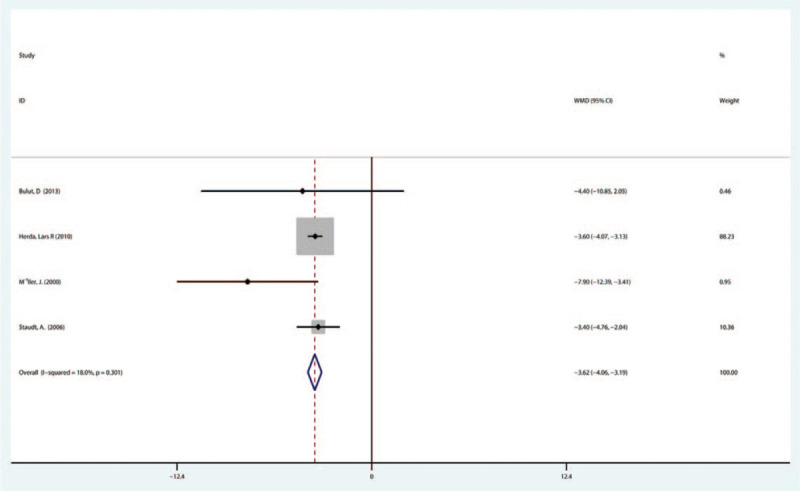
Forest plot showing the LVEDD immunoadsorption treatment in patients suffering from DCM. DCM = dilated cardiomyopathy, LVEDD = left ventricular end diastolic diameter.

Four studies involving 145 patients presented the LVEDD data and the result of the meta-analyses indicated that IA therapy was associated with significantly improved LVEDD (–3.62, 95% CI [–4.06 to –3.19]). In addition, there was no statistical heterogeneity (*P* = .301, *I*^*2*^ = 18.0%) (Fig. 4), as revealed by heterogeneity test.

#### 3.3.3 NYHA functional classification

**Figure 5:**
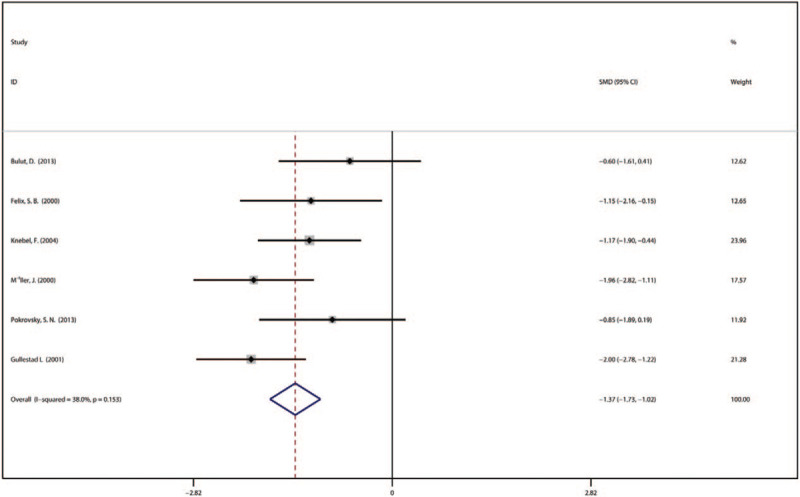
Forest plot showing the NYHA functional classification immunoadsorption treatment in patients of DCM. DCM = dilated cardiomyopathy, NYHA = New York Heart Association.

Six studies involving 144 participants presented the NYHA functional classification data. Patients in the IA group had relieved DCM symptoms according to the NYHA functional classification (–1.37, 95% CI [–1.73 to –1.02]) as compared with that of control group and the heterogeneity test showed no significant heterogeneity (*P* = .153, *I*^2^ = 38%) (Fig. 5).

#### 3.3.4 Safety

**Figure 6:**
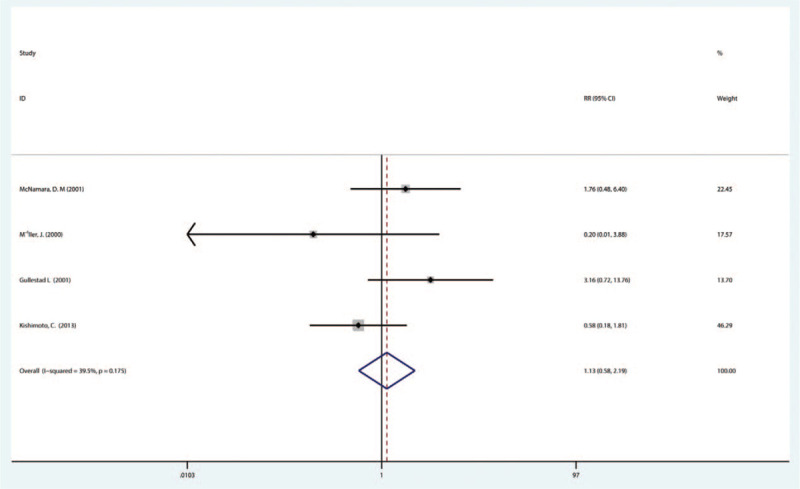
Forest plot showing the safety immunoadsorption treatment in patients of DCM. DCM = dilated cardiomyopathy.

Four studies reported adverse events during IA therapy. We found that IA was not significantly associated with any adverse event as compared with control (1.13, 95% CI [0.58–2.19]), and had no significant heterogeneity (*P* = .175, *I*^2^ = 39.5%) (Fig. 6).

#### 3.3.5 Publication bias

**Figure 7:**
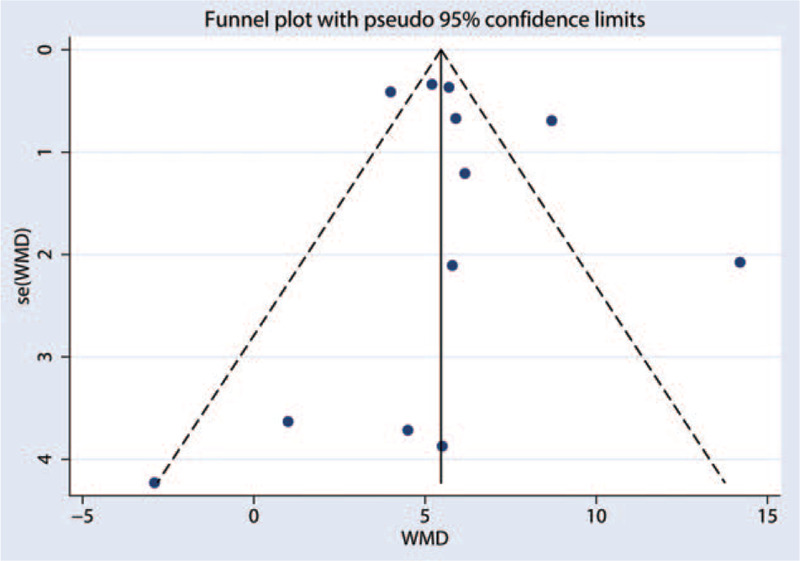
Funnel plot showing the analysis for publication bias for the LVEF of immunoadsorption treatment. LVEF = left ventricular ejection fraction.

To examine the bias of the meta-analysis publication, we used Funnel plot and Egger test. As shown in Fig. 7, LVEF Egger test (*P* = .59, 95% CI [–1.78–2.97]), LVEDD Egger test (*P* = .373, 95% CI [–4.09–2.38]), NYHA functional classification Egger test (*P* = .25, 95% CI [–4.93–14.39]), safety Egger test (*P* = .99, 95% CI [–15.10–15.00]).

## 4 Discussion

The present meta-analysis highlights the efficacy and safety of IA therapy in DCM patients and finds that IA therapy has improved LVEF, reduced LVEDD, and relieved DCM symptoms according to the NYHA functional classification in patients with DCM. Moreover, IA followed by IVIG substitution treatment also substantially improved LVEF as compared with that of control group, while IVIG only treatment failed to do so. However, the safety of IA therapy requires further confirmation. Overall, studies revealed beneficial effects of IA therapy in improving cardiac function and clinical outcomes in DCM patients. Recent studies have also shown that IA therapy can facilitate remodeling of left ventricle.^[23]^

The main pathological character of DCM is the remodeling of cardiac chambers. Ventricular remodeling is a dynamic process. During ventricular remodeling, the profiles of gene expression and protein composition are changed in response to IA therapy. Indeed, expression of immune activation and heart failure markers was increased in DCM patients, suggesting that increased immune response may accelerate the symptom onset of DCM. As the key surrogate measure of ventricular remodeling, LVEF has been used for evaluation of cardiac function, and is an important predictor for risk stratification,^[24]^ mortality, and rehospitalization in patients with left ventricular dysfunction. In this study, IA therapy is associated with improved LVEF, and there are significant subgroup interactions in IA and IA/IgG group but not in IVIG subgroup with high heterogeneity. The heterogeneity may derive from different characteristics of patients, study design, treatment duration, and follow-up time. Gullestad et al^[20]^ and McNamara et al^[22]^ find the association between IVIG and improved cardiac function. A statistically significant improvement in LVEF and quality of life, measured with standardized symptom assessments, has be seen at 3- and 6-months base on Guideline.^[25]^ However, more research is still needed to further confirm the efficiency and safety of IA therapy.

Emerging evidence has pointed out the role of immune system in cardiovascular diseases. Activation of the humoral immunity can result in production of circulating cardiac autoantibodies.^[26]^ Multiple cardiac autoantibodies have been identified in patients with myocarditis and DCM. Among DCM patients, various cardiac cellular proteins have been identified as antigens including the cardiac myosin, the beta-receptor, the Ca^2+^ channel, the M2 muscarinergic receptor, the Ca^2+^ ATPase, and the adenine nucleotide translocator.^[3]^ Clinical observation studies suggest that the development of effective IA is essential for protection against cardiac autoantibodies and is required to reduce the myocardial inflammation.^[26–28]^ Data obtained in this study indicate that IA and subsequent IgG substitution may represent an additional therapeutic tool for stabilization of the cardiovascular function of patients with severe heart failure due to DCM.

Studies showed that the IA treatment responders (improvement of LVEF ≥20% relative and ≥5% absolute) and non-responders showed variability in improvements on the cardiac function.^[29,30]^ The non-responders after IA/IgG treatment showed no significant improvement in cardiac function compared with responders, and the expression of fibrosis proteins were elevated with no significant changes in the gene expression. Certain cardiac antibodies, inflammatory markers, and activated T-cells might also be involved in cardiac dysfunction and that might explain why the IVIG only supplements were not effective on patients with DCM. Studies have shown that specific cardiac antibodies adsorption column gave better results than conventional IA.^[31,32]^ These studies focused on the blood signatures of responders and non-responders and could be used in the treatment of DCM in the future.

At the same time, our study has limitations on result interpretation of the meta-analysis. Firstly, the number of studies and participants was limited, and only 2 studies had >50 patients. Owing to small sample size we did not conduct further analyses to determine the source of heterogeneity and that could lead to exaggerated effects. Secondly, significant heterogeneity existed among studies and this might derived from diverse clinical characteristics, different treatments such as only IA or IA and subsequent IVIG substitution and treatment duration. Finally, the cardiovascular system related safety of IA should be further investigated.

## 5 Conclusions

Results of this meta-analysis revealed that IA improved LVEF, reduced LVEDD, and improved clinical outcome based on NYHA functional classification in DCM patients but the safety of IA still needs to be assessed. Overall, multi-center, double blinded studies are needed to precisely evaluate the effect of IA treatment in patients of DCM.

## Author contributions

**Conceptualization:** Ru-tao Bian, Zhen-tao Wang.

**Data curation:** Ru-tao Bian, Zhen-tao Wang, Wei-yu Li.

**Formal analysis:** Ru-tao Bian, Zhen-tao Wang, Wei-yu Li.

**Methodology:** Ru-tao Bian, Zhen-tao Wang, Wei-yu Li.

**Project administration:** Ru-tao Bian, Zhen-tao Wang.

**Software:** Ru-tao Bian, Wei-yu Li.

**Supervision:** Ru-tao Bian, Wei-yu Li, Zhen-tao Wang.

**Visualization:** Ru-tao Bian, Zhen-tao Wang, Wei-yu Li.

**Writing – original draft:** Ru-tao Bian, Wei-yu Li.

**Writing – review & editing:** Ru-tao Bian, Zhen-tao Wang, Wei-yu Li.
